# HCV Eradication with Direct-Acting Antivirals Does Not Impact HCC Progression on the Waiting List or HCC Recurrence after Liver Transplantation

**DOI:** 10.1155/2019/2509059

**Published:** 2019-01-17

**Authors:** Juliet A. Emamaullee, Mariusz Bral, Glenda Meeberg, Aldo J. Montano-Loza, Vincent G. Bain, Kelly Warren Burak, David Bigam, A. M. James Shapiro, Norman Kneteman

**Affiliations:** ^1^Division of Transplantation, Department of Surgery, University of Alberta, Edmonton, AB, Canada; ^2^Division of Abdominal Organ Transplantation, Department of Surgery, University of Southern California, Los Angeles, CA, USA; ^3^Division of Gastroenterology & Liver Unit, University of Alberta, Edmonton, AB, Canada; ^4^Division of Gastroenterology & Hepatology, University of Calgary, Calgary, AB, Canada

## Abstract

**Background:**

The introduction of direct-acting antivirals (DAA) for HCV has led to high rates of HCV eradication. Treatment of patients awaiting liver transplantation (LT) has been controversial. Recent data suggests that DAA treatment may accelerate recurrent HCC. The impact of DAA on delisting for HCC progression or recurrent HCC post-LT has not been well characterized.

**Methods:**

A retrospective review of both waitlist patients and LT recipients at a single institution was performed. Patient demographics, HCV treatment, HCC features and treatments, biopsy results, and graft and patient survival were evaluated. Patients on the LT waitlist or who were transplanted between January 2014 and December 2015 were included. Data was collected through December 2017 to have a minimum of two years of follow-up.

**Results:**

In the study period, 128 adult LT were performed. 44 patients were HCV+, and 68.2% (N=30) also had HCC. 38.6% (N=17) of HCV+ patients received DAA pre-LT, and 94.1% (N=16/17) achieved sustained virologic response (SVR) pre-LT. Among untreated HCV+ patients who underwent LT, 81.5% (N=22/27) received DAA post-LT, with 82.6% achieving SVR post-LT (N=18/22). 82.1% (N=23/28) of untreated post-LT patients underwent liver biopsy prior to therapy, and 52.2% had at least F1 METAVIR fibrosis. 87.5% (N=14/16) of active waitlist patients received DAA and achieved SVR. HCV eradication did not result in higher rates of delisting for HCC progression. Due to local HCC listing criteria of total tumor volume and AFP, 60% (N=18/30) of HCV+/HCC patients were beyond Milan criteria at the time of LT. Despite this, there was no difference in HCC recurrence rates post-LT, whether patients achieved SVR pre- or post-LT.

**Conclusions:**

These data suggest that HCV eradication pre-LT does not significantly impact waitlist time for HCV+ patients with HCC. HCV eradication does not impact rates of delisting for HCC progression or rates of HCC recurrence post-LT.

## 1. Introduction

Hepatitis C virus (HCV) infection continues to create a significant global health burden, with an estimated 185 million carriers worldwide. Despite efforts to treat HCV over the past twenty years, it is predicted that up to 45% of the more than three million patients with chronic infection in the US will go on to develop cirrhosis in the coming decade [1]. HCV-induced cirrhosis and HCV-associated hepatocellular carcinoma (HCC) continue to be leading indications for liver transplantation (LT) in the US and Canada [2, 3]. Recent advances in the development of direct-acting antiviral (DAA) treatment of HCV have led to high rates of HCV eradication, even in patients with decompensated cirrhosis [4]. The cornerstone of the first available DAA combination treatment, sofosbuvir (Sovaldi, Gilead Sciences, Inc.), a once-daily oral nucleotide analogue polymerase inhibitor, was approved in the US and Canada in December 2013 for the treatment of chronic HCV infection.

The cost associated with HCV eradication can range from $40,000 to 80,000 USD or more per patient depending on HCV genotype and recommended duration of therapy [5]. Treatment of patients with cirrhosis awaiting LT has not been routinely available, due to the high cost of these therapies and lack of established safety and efficacy data in this population. However, recent data has shown that DAA regimens such as sofosbuvir and velpatasvir can result in very high rates of sustained virologic response (SVR) in 78-94% in patients with decompensated cirrhosis, depending on the HCV genotype [4, 6]. However, there has also been data to suggest that patients can be treated effectively post-LT [7]. There has been speculation that HCV eradication pre-LT may result in improvement in a patient's severity of end-stage liver disease, resulting in a decrease in Model of End Stage Liver Disease (MELD) score, which could in theory disenfranchise some patients on the waiting list [8]. In some, it might obviate the need for a LT, but this appears to be the minority [9]. More recently, there has been data to suggest that HCV eradication with DAA may accelerate early recurrent HCC [10–12]. For these reasons, the optimal timing of HCV eradication in LT candidates continues to be actively debated.

In this study, a retrospective review of HCV+ patients in a single institution was performed, examining rates of HCV eradication pre- and post-LT and the impact of persistent HCV infection on post-LT outcomes. The effect of HCV eradication with DAA in patients with HCC was also studied, including liver transplant recipients and patients on the transplant waiting list.

## 2. Patients and Methods

### 2.1. Inclusion Criteria

This study included all adult patients who were transplanted at the University of Alberta, Edmonton, Alberta, Canada, with a diagnosis of HCV-induced cirrhosis between January 2014 and December 2015. This study period was selected to reflect the approval of sofosbuvir by Health Canada and the Food and Drug Administration, which occurred in December 2013. The study period was ended in December 2015 to provide at least two years of follow-up for review. Similarly, all patients on the LT waiting list with a  diagnosis of HCV-induced cirrhosis during the study  period were included, excluding those who received LT.  This included patients who were delisted during  the study period.  December 31, 2017, was the final follow-up date, and patients were censored as of this date for survival analyses. Adult patients on the waiting list or who received a LT in the study period but did not have a diagnosis of HCV-induced cirrhosis served as controls. For purposes of survival analysis, overall patient survival and HCC recurrence-free survival of patients transplanted from 2007 to 2013 were also evaluated. The University of Alberta Institutional Health Ethics Review Board approved this study.

### 2.2. Exclusion Criteria

This study excluded patients transplanted or on the transplant waiting list who were less than 18 years old, or those transplanted after December 2015. Patients who received multiorgan transplants (e.g., multivisceral or liver-kidney) were also excluded.

### 2.3. Clinical and Laboratory Assessments

Data collected included the following patient demographics: gender, age, weight, height, body mass index, blood group, etiology of cirrhosis, MELD-Na score at activation and transplant, and waitlist MELD (which would include any MELD exception points) at activation and transplant, if applicable. At the University of Alberta, patients with HCC receive MELD exception (eMELD) points for multifocal HCC or viable tumor(s) on imaging >2 cm in diameter, starting with an eMELD of 22 and increasing by 2 points every 3 months. Time on the waiting list was also evaluated, which was defined as the time between activation and LT, or the final date of the study period for waitlist patients. For HCV+ patients, viral genotype and antiviral treatment (type, duration, and response to treatment with SVR at 12 weeks) was recorded. Clinical values analyzed included liver biochemistries, serum creatinine, international normalized ratio, type of immunosuppression, and liver biopsy results. Among patients who received a LT, graft and patient survival were analyzed. For patients with a diagnosis of HCC, alpha fetoprotein (AFP), total tumor volume (TTV), number of tumors, and pre-LT HCC treatments were collected. Characteristics of liver explants from patients with HCC that were evaluated included number of tumors, TTV, histological grade, and the presence or absence of microvascular invasion. In our center, basiliximab induction combined with tacrolimus and mycophenolate mofetil based maintenance (steroid free) immunosuppression regimen is the standard of care. Patients with HCC are converted to sirolimus, where possible, at one month post-LT.

### 2.4. Statistical Analyses

The Fisher exact test was used to compare categorical variables, and the t-test was used to compare differences in means of continuous variables. Nonparametric variables in independent samples were compared by the Mann-Whitney test. An analysis of variance (ANOVA) was used to compare differences in means in patient demographics and tumor characteristics. HCC recurrence-free survival and overall patient survival were calculated with the Kaplan-Meier method and compared via the log-rank test. A P value <0.05 was considered to be significant. Statistical analysis was performed with SPSS Version 23.

## 3. Results

### 3.1. Study Population

In the study period, 128 adult patients underwent LT at the University of Alberta. Within this cohort, there were 44 HCV+ patients, and the majority were infected with HCV genotype 1 (Table 1; Supplemental Figure 1). When compared to the control group of recipients, HCV+ recipients were older with a mean age of 59.6±5.0 years, versus 49.3±13.9 years in the control group (p<0.001). There were a greater proportion of male recipients in both groups. The natural MELD-Na of HCV+ recipients was significantly lower than the control group, both at the time of activation (16.9±7.4 vs. 22.1±8.2; p=0.001) and at the time of LT (20.4±8.3 vs. 24.4±9.2; p=0.018). This can be attributed to the relatively high frequency of HCV+ recipients with concomitant HCC (68%), versus only 19% in the control group. Consequently, when comparing waitlist MELD, which includes HCC exception points, at the time of activation and at the time of LT, there was no difference between HCV+ recipients and controls.

When compared to the control group, HCV+ recipients on average had a longer waiting time prior to LT (410.6±504.6 vs. 225.9±28.8 days; p=0.007). Treatment of HCV+ patients on the waiting list with DAA and subsequent SVR did not alter the mean waiting time for LT when compared to untreated HCV+ patients (Table 1).

### 3.2. HCV Eradication in Liver Transplant Recipients

**Figure 1:**
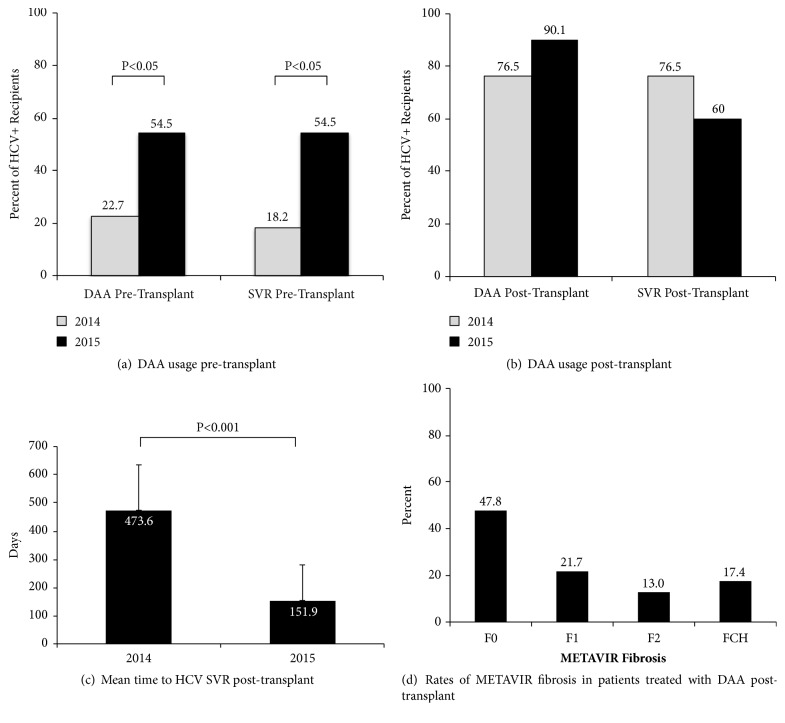
**DAA therapy pre- and post-liver transplantation. **There was a significant increase in pre-transplant utilization of DAA therapy between 2014 (N=5 patients treated pre-LT) and 2015 (N=12 LT patients treated pre-LT) (panel (a)). Among those treated with DAA pre-LT in 2014, the rate of SVR was 80%, with only 18.1% (N=4/22) of all patients with SVR pre-LT. In 2015, 100% of N=12 patients treated pre-LT achieved SVR, representing 54.5% of all patients transplanted in 2015. A high proportion of patients who were untreated at the time of transplant (N=17 patients in 2014 and N=11 patients in 2015) were treated with DAA post-LT, with 100% of those patients achieving SVR post-LT in 2014 and 60% achieving SVR post-LT in 2015 (panel (b)). The mean time to SVR significantly improved between 2014 and 2015 (c). A high proportion of untreated post-LT patients (N=23/28) underwent biopsy, and N=12/23 patients had at least F1 METAVIR fibrosis prior to starting DAA. Data are presented as mean ± SD.

In 2014, the first year following sofosbuvir approval, a modest proportion of HCV+ LT recipients were treated with DAA pre-LT (22.7%, N=5), with 80.2% of those treated achieving SVR (Figure 1(a)). Overall, only 18.2% (N=4/22) of the HCV+ transplant recipients received DAA and had SVR pre-LT in 2014. However, by 2015, there was a significant increase in the proportion of patients treated with DAA pre-LT, with 54.5% (N=12/22) of HCV+ transplant recipients being treated pre-LT and 100% of those treated achieving SVR (Figure 1(a); p<0.05 vs. 2014). Within our program, there has also been a concerted effort to treat HCV+ patients in the post-LT period, as shown in Figure 1(b). For patients transplanted in 2014, 76.5% (N=13/17) of patients with untreated HCV+ at the time of transplant received DAA post-LT, with 100% of those treated achieving SVR. Obtaining drug coverage in the post-LT period has been challenging in the Canadian healthcare environment, and as a result of treatment delays, 82.1% (N=23/28) of patients underwent percutaneous biopsy for abnormal liver function tests prior to receiving DAA. Among untreated HCV+ transplant recipients who underwent biopsy, 52.1% had at least F1 fibrosis according to the METAVIR scale (Figure 1(d)). There were four cases of biopsy-proven fibrosing cholestatic hepatitis (FCH), and two of these untreated patients died as a result at days 88 and 125 post-LT, respectively, despite the introduction of DAAs once the diagnosis of FCH was established. On average, patients transplanted in 2014 did not achieve SVR until 473.6 days, which improved to 151.9 days for patients transplanted in 2015 (Figure 1(c), p<0.001).

### 3.3. HCV Eradication on the Liver Transplant Waiting List

As of December 2015, there were 68 adult patients on the LT waiting list, with 18 HCV+ patients and 9 HCV+ patients with HCC. When compared to controls, a higher proportion of HCV+ patients were male (Table 2; 88.9% vs. 54.0%, p=0.009). There was no difference in natural MELD-Na or waitlist MELD between HCV+ patients and controls. The majority of HCV+ patients were infected with genotype 1, and 88.9% (N=16/18) had been treated with DAA, resulting in 87.5% SVR (N=14/16; Table 2). Among patients that did not achieve SVR, there was one patient with HCV genotype 4 and a natural MELD-Na of 21 who failed following 24 weeks of sofosbuvir/ribavirin. There was a second patient with HCV genotype 1 who failed to achieve SVR following 12 weeks of sofosbuvir and ribavirin. Two patients with HCV genotype 3 were intentionally not treated prior to LT, as a decision was made to wait for approval for a drug combination that had a high efficacy for genotype 3, which was difficult to eradicate in the early DAA era.

With emerging data to suggest that HCV eradication with DAA can accelerate recurrent HCC in non-transplant patients, patients who were delisted during the study period were carefully examined (Table 3). There were 43 HCV+ patients, and 22 of these also had HCC. Reasons for delisting are summarized in Table 3. There was no difference in rates of DAA treatment with subsequent SVR for patients with HCV, versus those with HCV and HCC (19.0% vs. 27.3%, p=0.523). Thirteen patients with HCV and HCC were delisted due to tumor progression, and an additional patient who was delisted for clinical deterioration also had evidence of HCC progression. This is similar to rates reported in a multi-institutional consortium of centers using total tumour volume (TTV) as a listing criterion [13]. No differences were observed in HCC characteristics (AFP, TTV, number of tumors, and days between last HCC treatment and delisting) between HCV+ and non-HCV patients. There was no difference in the proportion of patients with HCC progression with untreated HCV (N=11/16, 68.8%) versus those with SVR (N=3/6, 50% of HCV+/HCC waitlist patients with SVR). Treatment with DAA did confer a survival benefit to delisted patients, as 88.5% (N=23/26) of untreated delisted patients died, versus only 45.5% (N=5/13) of patients who achieved SVR and were delisted (Table 3, P=0.022).

### 3.4. HCV Eradication and Its Impact on HCC Recurrence

Given the controversy surrounding the potential impact of HCV eradication with DAA on HCC recurrence, HCV+ transplant recipients with HCC were also examined in detail (Table 4). The Canadian consensus criteria for HCC in LT are based on TTV <115 cm^3^ and AFP <400 ng/mL [13, 14]. As a result, patients with multiple small tumors, who would otherwise be outside of Milan criteria, are routinely transplanted with equivalent outcomes [13]. During the study period, 30 out of 46 patients transplanted for HCC were HCV+. Transplant recipients were beyond Milan criteria in 60% (N=12/18) of HCV+ patients and 50% (N=8/16) of control patients. HCV+ patients were significantly more likely to be beyond UCSF criteria when compared to control patients (53.3% vs. 25.0%, P=0.041). There was no difference between HCV+ patients and controls in terms of mean AFP, number of tumors, or TTV at the time of listing or in the last AFP/TTV prior to LT. Similarly, there was no difference between HCV+ patients and controls in liver explant characteristics, specifically in Milan criteria, number of tumors, TTV, histological grade, or presence of microvascular invasion. Pre-transplant therapies targeted towards HCC were also similar between groups.

It is our institutional practice to initiate mTOR-inhibitor based immunosuppression (sirolimus) regimens beginning one month post-LT in patients with HCC [15, 16]. The proportion of patients who were on sirolimus at two months and one year post-LT was similar in HCV+ patients and controls (Table 4).

**Figure 2:**
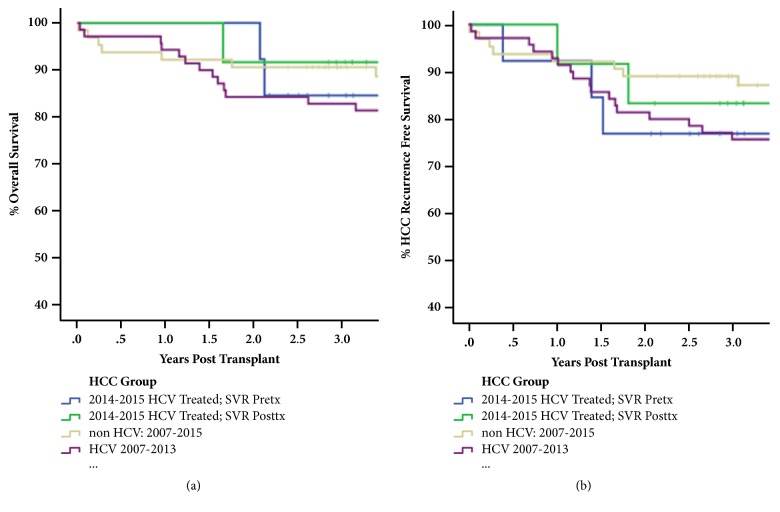
**HCV eradication with DAA does not significantly impact patient survival or HCC recurrence rates post-transplant.** There was no difference in patient survival between HCV+/HCC LT patients who achieved SVR pre-transplant (blue line, N=13), HCV+/HCC LT patients who achieved SVR post-transplant (green line, N=12), pre-DAA era HCV+/HCC LT patients (purple line, N=70), and HCC (non-HCV) LT patients (yellow line, N=63) (panel (a)). There was no difference in rates of HCC recurrence between these four cohorts as well. Data were analyzed using the Kaplan-Meier method with log rank analysis.

Overall patient survival and HCC recurrence-free survival were examined in HCV+ patients and control patients transplanted for HCC, with at least two years of follow-up (Figure 2). HCV+ patients were further subcategorized into those who achieved SVR pre-LT (N=13) versus those that achieved SVR post-LT (N=12). These were compared to patients transplanted for HCC within our institution in the pre-DAA era, from 2007 to 2013, and stratified for HCV+ (N=70) and non-HCV recipients (N=63). As shown in Figure 2(a), there was no significant difference between these four cohorts, with an overall survival >80% at two years post-LT. There was 100% one-year survival in patients with HCC who completed treatment with DAA, either pre- or post-LT. In terms of HCC recurrence-free survival, there was no significant difference between HCV+ patients transplanted for HCC who achieved SVR pre- (N=3/13 with recurrent HCC) or post- LT (N=2/12 with recurrent HCC; Figure 2(b)). Each of the patients with recurrent HCC was beyond Milan criteria based on liver explant analysis. Regardless of treatment with DAA pre- or post-LT, recurrent HCC was most often within the lung (N=4/5 patients, Table 4). These data were similar to historical controls.

## 4. Discussion

This study represents the first North American, single institution experience characterizing the impact of HCV eradication with DAA on patients with HCV and HCC on the LT waiting list and in LT recipients [17]. These data demonstrate that HCV eradication with DAA prior to LT can result in SVR in a high proportion of patients on the waiting list and does not impact mean waiting time, particularly when a majority of the patients are receiving MELD exception points for HCC (Tables 1 and 2). Treatment of HCV+ patients without HCC can result in clinical improvement and subsequent delisting, which has been reported by Pascasio et al [18]. Within our own program, we have observed a precipitous drop in the number of LT for HCV cirrhosis in the absence of HCC, from a peak of 28% in 2007 to 1.5% (N=1 patient) in 2017 (unpublished data). As a result, challenges to obtaining post-LT DAA have become less of an issue. In the present study, we observed that delaying HCV treatment until the post-LT period resulted in a high frequency of percutaneous liver biopsy, with >50% of patients developing at least F1 METAVIR fibrosis (Figure 1). It can be challenging to obtain coverage for DAA post-LT in the Canadian healthcare environment, resulting in long treatment delays, albeit with improvement as DAA became more readily available (Figure 1(c)). There were four cases of FCH in untreated patients, resulting in two potentially preventable deaths in the early post-LT period. Taken together, these data support the concept of attempting HCV eradication with DAA prior to LT, particularly in patients with HCC who will qualify for MELD exception and where coverage for DAA post-transplant can be difficult to obtain.

There have been several recent publications addressing the impact of DAA on HCC recurrence in patients with HCV. The Barcelona group published a report involving 58 patients with HCC that had been treated with surgical resection, radiofrequency ablation (RFA), or transarterial chemoembolization (TACE) resulting in a “complete response” [11]. This study reported an unexpectedly high HCC recurrence rate of 27.6% within 6 months for patients who received DAA. Importantly, this study excluded LT recipients. Also, 10% of patients in this study received TACE as their only treatment for HCC, which is considered to be less likely to result in a potentially curative response when compared to resection or ablative interventions. In contrast, the ANRS Collaborative Study Group published data examining HCC recurrence following DAA treatment for HCV among three distinct patient cohorts, including one subset that involved LT recipients [12]. The first arm of this study examined 267 patients that had previously received treatment for HCC, with 189 patients who subsequently received DAA treatment and 78 untreated controls. They excluded patients with active HCC. There was no difference in recurrence rates between patients who had received DAA and control patients. The second cohort included HCV+ recipients who were followed prospectively for HCC, with a cumulative 5-year HCC incidence of 13.9%. Incidental HCC was treated with resection or percutaneous ablation, and there was no difference in recurrence rates between patients that had received DAA (N=13) and untreated controls (N=66). The final cohort involved patients who were transplanted for HCV, with evidence of post-LT HCV recurrence. There were 314 LT recipients with a history of HCC who received DAA post-LT, and there were only 7 patients with recurrent HCC. Thus, this study did not find any evidence to suggest that HCV eradication with DAA increases the rate of recurrent HCC. A third recent publication from Tokyo involved 27 patients treated with DAA following RFA, with comparison to 38 patients who received IFN-based therapy and 861 control patients [10]. They observed no difference in rates of HCC recurrence among HCV+ patients treated with DAA, but did not examine the impact of DAA in LT. Taken together, much controversy still exists regarding the impact of DAA on HCC recurrence.

The present study addresses this concept in several important areas. First, there is no evidence to suggest that HCV eradication with DAA while on the waiting list results in rapidly recurrent or progressive HCC, ultimately leading to delisting (Table 3). In fact, the majority of patients that were delisted for tumor progression had not been exposed to DAA. There was a survival benefit to HCV eradication among patients who were delisted, which is in keeping with the findings of Belli* et al.*, who have demonstrated that a proportion of patients can improve enough following SVR to obviate the need for LT [9]. It is also possible that this is the consequence of patient selection bias, as those who were not offered DAA were in a worse overall clinical condition and/or had a history of noncompliance and were deemed to be poor candidates for DAA therapy.

Second, to our knowledge, this study represents one of the first examinations of the impact of DAA on LT recipients with HCC, both pre- and post-LT. Yang* et al*. reported the combined results from Mayo-Rochester and Mayo-Arizona, which showed that patients with HCC in the setting of HCV who achieved SVR pre-transplant (N=28) had a trend towards longer waiting times and were more likely to be beyond Milan criteria in their explants [19]. They also reported a trend towards earlier HCC recurrence in these patients, although it was not statistically significant (p=0.06). They did not compare these results to patients treated with DAA post-transplant. One unique aspect of our study is the Canadian HCC criteria for LT, which include AFP<400 ng/mL and a total tumor volume <115 cm^3^, which approximately equates one tumor approximately 6.1 cm in diameter or several smaller tumors. Importantly, it does not factor in total number of tumors and as a result many patients are technically beyond Milan or UCSF criteria. One might argue that these patients have a relatively higher risk of HCC recurrence overall, with >60% of HCV+/HCC transplant recipients being beyond Milan criteria in their explant (Table 4). Despite this, there was no difference in overall or HCC recurrence-free survival, whether DAA were utilized pre- or post-LT and when compared to historical HCV and non-HCV control recipients transplanted for HCC from the same institution, with > 2 years of post-transplant follow-up (Figure 2). One factor which may have a measurable impact on the relatively low incidence of recurrent HCC in our institution, despite having a majority of patients who would be considered “high risk” due to being beyond Milan criteria, is the implementation of early mTOR-inhibitor based immunosuppression. A recent multicenter, prospective randomized control trial has reported that sirolimus confers an overall and recurrence-free survival benefit in some lower risk patients with HCC [15].

There are limitations to this study. Data was collected retrospectively without intention to treat and as a result is relatively heterogeneous in terms of DAA treatment timing and strategy (Table S1). However, we are fortunate to have access to a provincial electronic medical record, which provides access to laboratory results, imaging, hospital admissions, microbiology including HCV PCR and genotype, prescribing information, and date of death that may otherwise not be captured. HCC characteristics, as well as pre-LT interventions, were also analyzed (Table 4). The overall cohort of LT recipients with HCV and HCC was relatively small with total N=30. However, if there was truly a high rate of HCC recurrence for patients treated with DAA, one would expect that it would be even more evident in the present study, where the majority of patients had active HCC at the time of LT, were beyond Milan criteria, and subsequently received immunosuppression. In all, 5 patients who received DAA in the setting of LT had recurrent HCC, which represents a two-year recurrence rate of 20%. This is in keeping with previous reports including a recent meta-analysis [20, 21]. It is also important to note that the patients treated with DAA in the present study received the drug in the early post-LT period, while those in previous reports received the drug at least one year or more post-LT [7, 12]. A recent publication by Sasaki* et al*. has reported that HCV clearance with DAA results in normalization of proinflammatory cytokine levels including IL-1*β*, tumor-necrosis factor-*α*, and IL-6 [22]. It is possible that changes in the overall immune milieu post-DAA allow for antitumor immune responses to be more effective, resulting in less HCC recurrence or progression [21, 22].

In summary, this study supports the strategy that HCV eradication can be attempted prior to LT in patients with HCC, with no apparent increase risk of HCC recurrence. This study has confirmed other larger study results demonstrating that DAA are effective in patients with decompensated cirrhosis, even those on the transplant waiting list [23]. HCV eradication may stabilize some patients on the waiting list, negating the need for LT, which is in keeping with the results of other larger studies [9]. Depending on regional variation, access to organs, and center-specific practice patterns, the decision to treat HCV+ recipients on the waiting list continues to be a complex topic; that being said, we saw no detriment to treating pre-LT patients at our institution. Finally, HCV eradication pre-LT should completely prevent the devastating complication of FCH, which has been reported in up to 10% of HCV+ LT recipients and was the observed rate in the present study [24]. This should provide a cost-savings to the healthcare system, as these patients often suffer from prolonged hospitalizations. Also, in the present outcomes-driven practice climate, prevention of even one or two post-LT mortalities is likely to result in a measurable improvement in one-year survival rates.

Some physicians may be resistant to initiating DAA therapy in HCV+ patients with HCC, especially given the recent controversial findings as outlined above. This study suggests that DAA treatment does not result in higher rates of delisting for tumor progression, nor impact HCC recurrence even in patients that are considered to be high risk (i.e., beyond Milan), even in this somewhat small retrospective cohort. As well, pre-LT HCV eradication allows for aggressive treatment of rejection episodes post-LT, without concern of accelerating the natural history of recurrent HCV or precipitating FCH in the setting of corticosteroid use. For these reasons, we have made an effort to treat all HCV+ patients prior to LT in our own institution.

In conclusion, this study demonstrates that HCV eradication can be achieved in a majority of patients with HCC pre-LT, does not result in longer waiting times or a high proportion of treatment failures, and does not impact rates of delisting for HCC progression or rates of HCC recurrence post-LT.

## Figures and Tables

**Table 1:** Transplanted patient demographics.

	**HCV (N=44)**	**Non HCV (N=84)**	**P Value**
**Age, mean±SD [range]**	59.6±5.0	49.3± 13.9	P<0.001
	[43.3-67.9]	[18.9-69.9]	
**Gender, N=Male (**%** Male)**	35 (79.6)	55 (65.5)	P=0.099
**BMI at Transplant, mean±SD [range]**	27.9±4.9	27.3±6.1	P=0.552
	[19.7-39.3]	[18.1-47.2]	
**MELD-Na at Activation, mean±SD [range]**	16.9±7.4	22.1±8.2	P=0.001
	[6-35]	[7-46]	
**Waitlist MELD for Activation, mean±SD [range]**	21.4±6.0	23.5±7.0	P=0.109
	[9-35]	[9-46]	
**MELD-Na at Transplant, mean±SD [range]**	20.4±8.3	24.4±9.2	P=0.018
	[9-45]	[6-48]	
**Waitlist MELD at Transplant, mean±SD [range]**	26.4± 6.3	27.3±7.4	P=0.534
	[10-45]	[13-48]	
**Days on waitlist prior to transplant, mean±SD [range]**	410.6±504.6	225.9±28.8	P=0.007
	[0-2412]	[0-1254]	
** HCV SVR Pre-Transplant (N=17)**	492.0±619.1		
	[10-2412]		P=0.308
** Untreated Pre-Transplant (N=27)**	415.0±407.7		
	[0-1692]		
**HCV Genotype, N (**%**)**			
** 1**	34 (77.3)		
** 2**	2 (4.5)		
** 3**	7 (15.9)		
** 4**	1 (2.3)		
**Diagnosis, N (**%**)**			
** HCV Alone**	14 (31.8)		
** HCV & HCC **	30 (68.2)		
** HCC, underlying diagnosis [N]**		16 (19.0)	
** ETOH **[8]			
** HBV **[2]			
** NASH **[3]			
** PBC **[1]			
** PSC **[1]			
** Chronic cholestatic (PBC, PSC)**		23 (27.4)	
** Hepatocellular (Cryptogenic, AIH, HBV, NASH, ETOH)**		31 (36.9)	
** Metabolic (A1AT, Urea Cycle)**		4 (4.8)	
** Acute Failure (Seronegative Hepatitis, Tylenol Overdose)**		5 (6.0)	
** Other**		5 (6.0)	

**Table 2:** Patient demographics on liver transplant waitlist.

	**HCV (N=18)**	**Non HCV (N=50)**	**P Value**
**Age, mean±SD [range]**	54.9±7.0	48.8±12.6	P=0.055
	[37-62]	[21-66]	
**Gender, N=Male (**%** Male)**	16 (88.9)	27 (54.0)	P=0.009
**BMI at Activation, mean±SD [range]**	28.2±4.6	24.7±4.1	P=0.004
	[21.9-36.0]	[17.0-36.3]	
**MELD-Na as of 12/2015, mean±SD [range]**	16.0±5.9	15.0±5.9	P=0.270
	[6-23]	[6-29]	
**Waitlist MELD as of 12/2015, mean±SD [range]**	17.1±6.8	16.4±5.6	P=0.327
	[6-28]	[7-30]	
**Blood group, N (**%**)**			
A	7 (38.9)	21 (42)	
AB	0 (0.0)	1 (2)	
B	1 (5.6)	3 (6)	
O	10 (55.6)	25 (50)	
**HCV Genotype, N (**%**)**			
1	12 (66.7)		
2	2 (11.1)		
3	2 (11.1)		
4	2 (11.1)		
**HCV Treatment, N (**%**)**	17 (94.4)		
** **SVR, N (%)	13 (76.5)		
**Diagnosis, N (**%**) **			
HCV Alone	9 (50)		
HCV & HCC	9 (50)		
HCC, underlying diagnosis (N)		6 (12)	
HBV (5)			
AIH (1)			
Chronic cholestatic (PBC, PSC)		17 (34)	
Hepatocellular (ETOH, AIH, Cryptogenic, HBV, NASH)		23 (46)	
Metabolic (A1AT, MSUD)		2 (4)	
Acute failure (Seronegative hepatitis)		2 (4)	

**Table 3:** Demographics of patients delisted in 2014-2015.

	**HCV (N=43)**	**Non-HCV (N=54)**	**P Value** **(subgroups)**
**Age, mean±SD, [range]**	55.9±5.3	45.5±20.2	
	[44-65]	[19-68]	
**Gender, N=Male (**%** Male)**	30 (69.8)	27 (50.0)	
**MELD-Na at listing, mean±SD [range]**	14.1±1.2	16.9±1.1	
	[6-41]	[6-42]	
**Blood group, N (**%**)**			
A	19 (44.2)	16 (29.6)	
AB	2 (4.7)	1 (1.8)	
B	5 (11.6)	8 (14.8)	
O	17 (39.5)	29 (53.7)	
**HCV Genotype, N (**%**)**			
1	27 (62.8)	N/A	
2	4 (9.3)		
3	11 (25.6)		
4	1 (2.3)		
**Reason for Delisting, N (**%**)**			
Noncompliance	9 (20.9)	10 (18.5)	
Death on waiting list	11 (25.6)	15 (27.7)	
Clinical deterioration/ medical contraindication	7(16.2)	15 (27.7)	
HCC tumor progression	13 (30.2)	3 (5.6)	
Clinical improvement	1 (2.3)	5 (9.3)	
Moved/transplanted elsewhere/other	2 (4.6)	6 (11.1)	
**Patients also with HCC, N (**%**)**	22 (51.1)	7 (12.9%)	
Listing AFP, mean±SD, [range]*∗*	23.5±28.9	10.1±7.1	
Listing TTV, mean±SD, [range]*∗*	9.0±12.3	4.2±5.2	
Listing # tumors, mean±SD, [range]*∗*	1.6±1.9	1.4±1.1	
Within Milan Criteria at Listing, N (%)*∗*	12/16 (75%)	5/5 (100%)	
Days between last HCC treatment and delisting, mean±SD, [range]*∗*	181.1±139.5	355.0±522.6*∗∗*	
**HCV patients with HCC, N (**%**)**			
Untreated HCV (% patients HCV/HCC)	16 (72.7)		
Patients with SVR (% patients with HCV/HCC)	6 (27.3)		
**HCV SVR, N (**%** of subgroup)**			
HCV alone, Total N=21	4 (19.0)		0.523
HCV/HCC, Total N=22	6 (27.3)		
**HCC progression, N (**%** of subgroup)**			
Untreated HCV, Total N=16	11 (68.8)		0.415
Patients with SVR, Total N=6	3 (50.0)		
**Death after delisting, N (**%** of subgroup)**			
Untreated HCV, Total N=26	23 (88.5)		0.022
Patients with SVR, Total N=13	5 (45.5)		

*∗*Data for this subgroup includes N=16 HCV/HCC patients and N= HCC (non-HCV) patients, as not all patients who were delisted have detailed HCC data in our registry. *∗∗*Two patients in the HCC (non-HCV) analysis did not receive HCC treatment between listing and delisting.

**Table 4:** HCC Characteristics for transplanted patients.

	**HCV (N=30)**	**Non-HCV** **(N=16)**	**P Value**
**Within Milan Pre-Transplant, N (%)**	12 (40.0)	8 (50.0)	P=0.525
**Within UCSF Pre-Transplant, N (%)**	14 (46.7)	12 (75.0)	P=0.041
**Listing:**			
AFP (ug/L), mean±SD [range]	17.3±23.5 [1-119]	8.8±14.0 [2-58]	P=0.211
Number of Tumors, mean±SD [range]	1.4±2.2 [0-10]	0.7±0.8 [0-2]	P=0.198
Total Tumor Volume (cm^3^), mean±SD [range]	6.4±10.8 [0-37.2]	5.4±7.5 [0-22.5]	P=0.761
**Last Values Prior to Transplant:**			
AFP(ug/L), mean±SD [range]	39.1±76.8 [2-347]	4.8±3.3 [1-14]	P=0.082
Total Tumor Volume (cm^3^), mean±SD [range]	7.3±16.9 [0-81.8]	4.3±10.0 [0-38.8]	P=0.524
**Explant Characteristics:**			
Within Milan, N (%)	19 (63.3)	11 (68.8)	P=0.721
Number of Tumors, mean±SD [range]	4.5±6.8 [0-35]	1.9±2.6 [0-10]	P=0.152
Total Tumor Volume (cm^3^), mean±SD [range]	14.5±27.2 [0-133.8]	19.9±31.9 [0-113.1]	P=0.550
Histological Grade, N (%)			
Necrotic	3 (10.0)	3 (18.8)	
Well differentiated	3 (10.0)	3 (18.8)	
Moderately differentiated	20 (66.7)	8 (50.0)	
Poorly differentiated	4 (13.3)	2 (12.5)	
Microvascular invasion, N (%)	3 (10.0)	1 (6.3)	
**Pre-Transplant Therapy, N (%)**			
None	5 (16.7)	4 (25.0)	
Alcohol Ablation	1 (3.3)	1 (6.3)	
Radiofrequency Ablation	16 (53.3)	9 (56.3)	
TACE	18 (60.0)	6 (37.5)	
SIRT	4 (13.3)	1 (6.3)	
Resection	0	2 (12.5)	
**mTOR Inhibitor Post-Transplant, N (%)**			
2 months post-transplant	19 (63.3)	9 (64.3)	P=0.386
1 year post-transplant	14 (51.9)	9 (60.0)	P=0.739
**HCC Recurrence among HCV+ LT Patients**			
Pre-LT SVR (N=3):			
Time to recurrence post-LT, days	144, 442, 521		
Location of recurrence	Multifocal, Lung, Lung		
HCC Treatment	Sorafenib, None, None		
Post-LT SVR (N=2):			
Time to recurrence post-LT, days	375, 674		
Location of recurrence	Lung, Liver Allograft		
HCC Treatment	None, RFA		

## Data Availability

The data used to support the findings of this study are available from the corresponding author upon request.

## Abbreviations

AFP: Alpha fetoprotein
DAA: Direct-acting antiviral
FCH: Fibrosing cholestatic hepatitis
HCC: Hepatocellular carcinoma
HCV: Hepatitis C virus
MELD: Model of end stage liver disease
RFA: Radiofrequency ablation
SIRT: Selective internal radiation therapy
SVR: Sustained virologic response
TTV: Total tumor volume
TACE: Transarterial chemoembolization.

## Supplementary Materials

Supplementary Materials Supplemental Figure 1: schematic outline of HCV+ LT patients included in the study. Supplemental Table 1: HCV treatment regimens.
