# Supplementary material for: HCV Eradication with Direct-Acting Antivirals Does Not Impact HCC Progression on the Waiting List or HCC Recurrence after Liver Transplantation
Source: Can J Gastroenterol Hepatol. 2019 Jan 17;2019:2509059. doi: 10.1155/2019/2509059 (PMC6354133; doi:10.1155/2019/2509059)
Supplement: Supplementary Materials — Supplemental Figure 1: schematic outline of HCV+ LT patients included in the study. Supplemental Table 1: HCV treatment regimens. [file 2509059.f1.pdf]

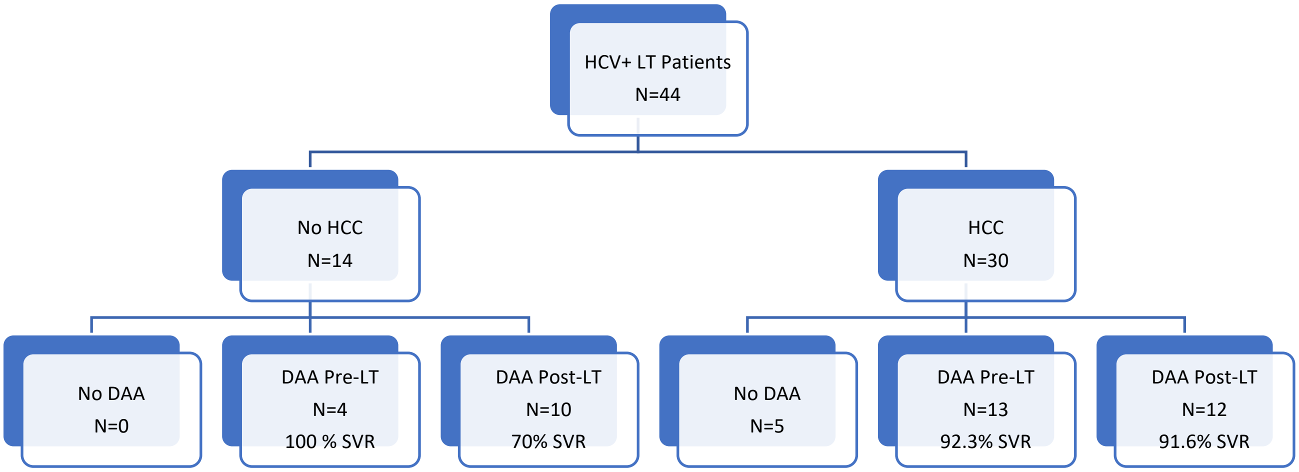

Supplemental Figure 1: Schematic outline of HCV+ LT patients included in the study.

Supplemental Table 1: HCV Treatment Regimens

Genotype, N (%)	DAA Pre-Transplant (N=17)	DAA Post-Transplant (N=23)	Patients without SVR Post-Transplant (N=8)
1			
Sofosbuvir/Ledipasvir	12 (70.6)	14 (60.9)**	4
Sofosbuvir/Ribavirin	1 (5.9)*	3 (13.0)	1-failed Sofosbuvir/Ledipasvir
Sofosbuvir/Simeprevir	1 (5.9)	2 (8.7)	1-death on treatment with FCH
			1-death on treatment with ductopenic rejection
			1-untreated
2			
Sofosbuvir/Ribavirin	1 (5.9)	0	0
Sofosbuvir/Ledipasvir	0	1 (4.3)	
3			
Sofosbuvir/Ribavirin	1 (5.9)	0	4
Sofosbuvir/Ledipasvir	0	1 (4.3)	3-untreated
Study Protocol	0	1 (4.3)***	1-death with FCH
4			
Sofosbuvir	1 (5.9)	0	0

*N=1 Failed to achieve SVR; **N=2 Failed to achieve SVR; ***Presently on treatment.
FCH (Fibrosing Cholestatic Hepatitis)
